# Health and development of ART conceived young adults: a study protocol for the follow-up of a cohort

**DOI:** 10.1186/1742-4755-10-15

**Published:** 2013-03-15

**Authors:** Cate Wilson, Karin Hammarberg, Fiona Bruinsma, Turi Berg, David Amor, Ann Sanson, Jane R Fisher, Jane Halliday

**Affiliations:** 1 Public Health Genetics, Murdoch Childrens Research Institute, 5th Floor, Royal Childrens Hospital, Flemington Road, Parkville 3052, Australia; 2 Jean Hailes Research Unit, Jean Hailes Research Unit, School of Public Health and Preventive Medicine, Monash University, Level 6 The Alfred Centre, 99 Commercial Rd, Melbourne, 3004, Australia; 3 Cancer Epidemiology Centre, Cancer Council Victoria, 100 Drummond Street, Carlton, 3053, Australia; 4 Clinical Genetics Research, Murdoch Childrens Research Institute, 4th Floor, Royal Childrens Hospital, Flemington Road, Parkville 3052, Australia; 5 Department of Paediatrics, University of Melbourne, Parkville, 3052, Australia; 6 Community Child Health, Murdoch Childrens Research Institute, Royal Children’s Hospital, Flemington Road, Parkville, 3052, Australia

**Keywords:** Assisted reproductive technology, In vitro fertilization, Health, Development, Psychological adjustment, Follow-up

## Abstract

**Background:**

Use of assisted reproductive technologies (ART) continues to increase, yet little is known of the longer term health of ART conceived offspring. There are some adverse birth outcomes associated with ART conception but the subsequent developmental trajectory is unclear. Undertaking research in this area is challenging due the sensitive nature of the topic and the time elapsed since birth of the ART conceived young adults. The aim of this report is to describe a research protocol, including design and ethical considerations, used to compare the physical and psychosocial health outcomes of ART conceived young adults aged 18-28 years, with their spontaneously conceived peers.

**Design:**

This is a retrospective cohort study of mothers who conceived with ART in Victoria, Australia and gave birth to a singleton child between 1982 and 1992. A current address for each mother was located and a letter of invitation to participate in the study was sent by registered mail. Participation involved completing a telephone interview about her young adult offspring’s health and development from birth to the present. Mothers were also asked for consent for the researcher to contact their son/daughter to invite them to complete a structured telephone interview about their physical and psychosocial health. A comparison group of women living in Victoria, Australia, who had given birth to a spontaneously conceived singleton child between 1982 and 1992 was recruited from the general population using random digit dialling. Data were collected from them and their young adult offspring in the same way. Regression analyses were used to evaluate relationships between ART exposure and health status, including birth defects, chronic health conditions, hospital admissions, growth and sexual development. Psychosocial wellbeing, parental relationships and educational achievement were also assessed. Factors associated with the age of disclosure of ART conception were explored with the ART group only.

**Discussion:**

The conceptualization and development of this large project posed a number of methodological, logistical and ethical challenges which we were able to overcome. The lessons we learnt can assist others who are investigating the long-term health implications for ART conceived offspring.

## Background

Since in vitro fertilisation (IVF) became available, around 5 million children have been born worldwide through assisted reproductive technologies (ART) [1]. Although ART is generally presumed to be safe, it is not known if the drugs used for ovarian stimulation, the manipulation of gametes, the artificial environment for fertilisation and the early embryo’s intrauterine exposure to hormones, create longer term health risks for offspring [2-6]. It may also be that the experiences of infertility and assisted conception impact on parenting style and parent-child relationships in ways that may affect the psychosocial development of the children [7-11].

### Evidence gaps and the significance of this work

Evidence of health outcomes for ART conceived children is accruing and indicate that, in spite of worse perinatal outcomes, their health and development is comparable to spontaneously conceived children’s[3,10,12-14]. However, very little is known about the health of people 18 years and older, largely due to the lack of adult ART-conceived populations [15]. Given the increasing use of ART worldwide, evidence about the long-term health effects of ART is of paramount importance and is required in order to inform people experiencing fertility difficulties, service planners, practitioners and policy makers[1,16].

### Study aims and hypotheses

The aim of this study was to compare the physical and mental health, educational achievements, and social development of young adults conceived through ART with spontaneously conceived young adults. The hypotheses were that:

1) The peri-conception environment and intrauterine exposures would lead to poorer physical health outcomes through epigenetic changes influencing growth and development and higher prevalence of chronic illness, compared to those conceived spontaneously.

2) Being conceived following parental infertility and assisted conception would lead to poorer outcomes in wellbeing and quality of life in young adults, compared to those conceived spontaneously.

## Methods/design

### Study design

The study was a comparison between a population based cohort of women and their ART conceived young adult offspring, with a cohort of women randomly selected from the general population of women in Victoria, Australia, and their spontaneously conceived young adult offspring, frequency matched on age and gender. This comparison group may include some subfertile mothers but this makes it a comparison group that truly reflects the general population. This is important when the study is trying to determine risk of adverse outcomes in offspring of the combined effect of infertility and ART in relation to the general population [17].

### Setting

The study setting was Melbourne IVF and Monash IVF infertility treatment centres located in Victoria, Australia. These two services were the primary ART treatment centres in Victoria during the period 1982 to 1992.

### Study timeline

A pilot study was conducted in 2007 to determine the feasibility and acceptability of approaching mothers up to two decades after cessation of ART treatment, and assess their recall of the health and development of their ART conceived young adult children [18]. This qualitative study concluded that with careful and sensitive recruitment strategies, it is feasible and acceptable to contact women to assess the health of their ART-conceived young adult offspring. Preparations for the main study commenced in 2008 with funding and ethics applications both involving consultation with parents of ART-conceived young adults and ART-conceived young adults themselves to ensure that the proposed recruitment strategies were perceived as achievable and the study materials relevant and acceptable. Recruitment and data collection took place between 2009 and 2011 and data analysis commenced in 2012.

### Exposed group

#### Mothers – eligibility criteria

Potential study participants were women who had ART and gave birth to one or more singleton children between January 1982 and December 1992. ART included in-vitro Fertilisation (IVF), gamete intrafallopian transfer (GIFT), tubal embryo stage transfer (TEST) or pronuclear stage transfer (PROST) in a stimulated or natural cycle; with own or donor gametes; using fresh or frozen embryos.

#### Exclusion criteria

Women were excluded if they were residing overseas, unable to be traced, had inadequate English to complete the interview, or who had died or whose child had died.

#### Young adults - eligibility criteria

Participants were the ART-conceived young adults aged 18 years or more, whose mother consented to the researchers approaching them.

#### Tracing of families

Maternal deaths were ascertained through electronic linkage with the Australian National Death Index (http://www.aihw.gov.au/national-death-index/). Offspring deaths were unable to be determined unless the ART service had been informed of this. Due to the time elapsed since the ART treatment, all maternal addresses provided at the time of treatment were checked for accuracy using electronic linkage with the Australian Electoral Commission (AEC) records (http://www.aec.gov.au/*)*. This yielded a current address for 72% of potential participants. Following manual tracing methods in the AEC or electronic telephone index, an additional 8% of potential participant’s addresses were identified. Overall, 80% of women were able to be located.

#### Recruitment procedures

A registered letter of invitation was sent to potential participants from a doctor at the ART centre where the woman had been treated. This letter provided a detailed explanation of the purpose of the study and what participation entailed. If there was no response, a reminder letter was sent four weeks later, allowing time for returns if the address was incorrect or the addressee did not collect the letter. Towards the end of the recruitment period, another letter was sent to all non-responding mothers to advise them that participation was still possible and that their contributions would be valuable. This permitted them a final opportunity to participate and meant that they received the final reminder between two and sixteen months after the original approach. Mothers were asked to return a form to the research team indicating their willingness to take part in the study or their decision to decline. Mothers’ consent for the researchers to contact their offspring and their daughter’s/son’s contact details were also sought at this stage. Both the mother and young adult were encouraged to take part individually but mothers who were unwilling to allow researchers to contact their offspring were given the option of taking part in the study on their own. Mothers with more than one ART conceived child in the study period were invited to complete a separate interview in relation to each child. With the mother’s consent and provision of contact details, young adults were telephoned, provided with detailed information about the study and invited to complete the study interview.

### Unexposed group

#### Mothers- eligibility criteria

Potential participants were women who had conceived spontaneously and given birth to a singleton child in Victoria between January 1982 and December 1992. Only one child born in this time period was included.

#### Exclusion criteria

Women were excluded if they had inadequate English for completion of the interview, or if their child had died.

#### Young adults – eligibility criteria

Participants were the spontaneously conceived, Australian born young adults aged 18 years or more, whose mother consented to the researchers approaching them.

#### Recruitment procedures

Households within Victoria were selected through random digit dialling and screened for eligible mothers by the Social Research Centre (SRC, http://www.srcentre.com.au). The SRC conducts, under contract, quantitative and qualitative research across all areas of social and health research for academic institutions, government, not for profit organisations and corporations. Women who agreed to be contacted by the research team were subsequently telephoned and given a detailed explanation of the purpose of the study and what participation entailed. With the mother’s consent and provision of contact details, young adults were telephoned, provided with detailed information about the study and invited to complete the study interview.

Participating mothers and young adults in the ART and control group each completed the telephone interview at a convenient time that allowed adequate privacy. Both were offered a summary of findings at the completion of the study and the young adults received a $25 gift voucher.

### Frequency matching of participants

**Figure 1:**
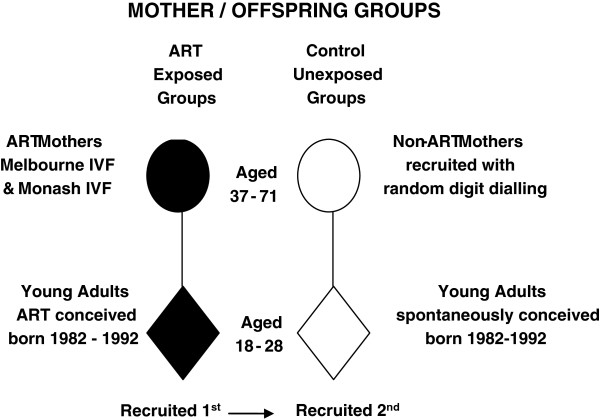
Study populations.

ART mothers were recruited first so that unexposed mothers could be proportionately recruited from similar geographic locations [19]. Young adults were subsequently recruited and similar proportions of unexposed males and females born within the 1982 -1992 period were recruited to frequency match the exposed young adults. There was no individual matching done (see Figure 1).

### Data sources

#### Structured interview schedule

Potential confounders and outcomes of interest were identified from the available evidence. The proposed outcomes were reviewed by members of the consumer advocacy group ‘AccessAustralia’ (http://www.access.org.au) for relevance and acceptability. Questions were piloted to test wording, flag sensitive areas and gauge the time taken to complete. The interviews, which included standardised measures and study-specific questions, were designed for a computer assisted telephone interview (CATI) system. The final maternal interview included 80 items and took approximately 35 minutes to complete. The young adult interview included 150 items and took approximately 30 minutes to complete (many questions were brief and able to be answered quickly). All interviews were administered by a small group of trained personnel at scheduled times. A protocol was devised for the interviewers to flag participants who seemed upset or depressed and who may have required subsequent support.

#### Medical records (exposed group only)

In order to compare characteristics of the non-participating ART mothers with the participating ART mothers, ethics approval was granted to obtain de-identified information from all mothers’ ART related medical records. Data were available on type of treatment, use of donor gametes and obstetric/perinatal outcomes, which had been reported to the ART clinic either by the treating obstetricians or the parents themselves and included: gestational age, birth weight, and birth defects. With consent from the participating mothers, additional data on infertility etiology, ovarian stimulation protocol, whether fresh or cryo-preserved embryos were used; age and number of embryos transferred; and number of sacs at ultrasound, were also obtained. Data were provided from hard copy or electronic data records using a standardised case report form.

### Outcomes

#### Primary outcome

The primary outcome of interest from the young adult interview was their self-reported quality of life reflected in physical, psychological, social, and environmental domains.

#### Secondary outcomes

Secondary outcomes from the maternal interview were her reports on child special health care needs, prevalence of birth defects, chronic health conditions and hospital admissions. Secondary outcomes from the young adult interview included their self-reported growth and sexual development, educational achievement, prevalence of chronic health conditions, hospitalisations, and quality of parental relationships.

### Confounders

Maternal age; parity; household income, perception of financial situation; marital status; maternal education; paternal occupation; family structure; residential location; type of infertility; obstetric complications; gestation at delivery; mode of delivery; offspring birth weight, gender; current age and relationship status; young adult employment.

### Measures

#### Perinatal outcomes (maternal report)

Data on perinatal outcomes included length of gestation and birth weight. Small or large for gestational age was defined as birth weight below or above the 10th percentile for gestational age respectively, using Australian population-based data for the calculation [20].

#### Birth defects (maternal report)

Mothers were asked to recall if their baby had any birth defects and to describe in detail the birth defect(s). These were coded as ‘major’, ‘minor’ or ‘not a birth defect’ using the Victorian Birth Defects Register classification system [21] and an International Classification of Diseases code (ICD10) was assigned [22]. The coding was performed blinded to the mode of conception and a paediatric expert was consulted throughout the coding process. Up to three defects were coded per individual (no-one exceeded this). Sometimes, congenital conditions were reported in response to the chronic illness or hospitalisation questions – in which case they were coded as above and added to the birth defects data e.g. hospitalisation for surgery for undescended testicles. The proportions with a major or minor defect and the proportion with related groups of birth defects (e.g. cardiovascular; gastrointestinal; genitourinary; musculoskeletal; genetic) were calculated.

#### Chronic health conditions (maternal report)

Data on chronic health conditions in offspring were collected from mothers using a question adapted from the Child Health Questionnaire [23,24]. Conditions qualified as a chronic illness if the condition had persisted over a year (including on and off) and the individual had consulted a health professional yearly or more often. The person’s age at onset and ceasing of the condition were recorded or if it was ongoing (or intermittent). An ICD10 code [22] was assigned to conditions and up to eight chronic conditions were coded per individual. Conditions that had arisen through injury or external consequences were not included.

The proportions with one or more chronic condition and the proportions with related types of conditions using ICD10 groupings were calculated. The proportion with one or more ongoing conditions was also analysed.

#### Hospital admissions (maternal report)

Study specific questions were used to ask mothers to report the number, reason and total length in days for offspring hospital admissions that occurred during the first year of life, pre-school period, primary school and secondary school period (up to 18 years). The condition causing the reported admissions was assigned an ICD10 code [22]. The proportions with one or more hospital admissions ever or with one or more admissions during school/age related periods (above) were calculated.

#### Child special health care needs screener (maternal report)

The mother-rated health of their child from 0 to 18 years was measured using the Child Special Health Care Needs (CSHCN) Screener to indicate use of health care for health concerns that persisted for a period of a year or more in the first 18 years of life [25]. The tool has five domains; need for prescription medication; need for more medical or mental health care or educational services than is usual for other children of the same age; limitations in doing things that are usual for age; need for physical, occupational or speech therapy; emotional or behavioural problems for which counselling is needed. The data items were coded, summed and scored as positive or negative according to Bethell [25] for each or the five domains. Unknown values within each domain (don’t know, refused, missing) were excluded from the denominator as per recommendations from the Child and Adolescent Health Measurement Initiative website [26].

#### WHOQol-Bref Australian version (young adult report)

The Australian WHOQol-Bref measures subjective quality of life [27]. This validated tool contains 26 items and demonstrates sensitivity to the health status of the respondent and sound psychometric properties. It has two general questions and four domains where the physical health, psychosocial, social, and environment domains have 7, 6, 3, and 8 items respectively. Each domain was coded, summed and scored according to prescribed methods to create a raw domain score [28]. Unknown values within each domain (don’t know, refused, missing) were imputed using horizontal mean imputation provided there were less than three missing items, as per the recommendations of Hawthorne [29]. Raw domain scores were then transformed to percentage scores.

#### Exercise behaviour – (young adult report)

Young adults were asked about the frequency of undertaking sport /exercise and vigorous exercise using validated questions from the Australian Temperament Project [30].

#### Measures of growth and development - (maternal and young adult report)

Young adult current height and weight data reported by young adults were used to generate a body mass index (BMI) measurement [weight(kg)/height(m)^2^]. Maternal and paternal height data collected from mothers were used to generate a ‘mid-parental height’ in order to adjust young adult height and BMI measurement for parental heights [31]. In the case of a donor gamete being used, no such calculation was made.

BMI measurements were also classified to determine underweight (<18.5 kg), normal weight (18.5-24.9 kg), overweight (25-29.9) and obesity (>=30 kg) [32].

Pubertal development was assessed from young adult report using the Adolescence Scale (AS-ICSM). This instrument has been found to be reliable in the recall of pubertal events and useful for the identification of maturational extremes [33]. It consists of two (female) or four (male) questions which ask the individual to recall their perceived rate of pubertal development in relation to their peers as well as their own pubertal milestones. The combined items enable a composite score to be created for a measure of relative maturational state independent of sex. The composite score is derived by summing the items and dividing by the number of items in the questionnaire for each sex. Missing values for each item were replaced with the mean of the z-score created for each of the puberty items for males and females according to Kaiser [34].

#### Sexual orientation (young adult report)

Young adults were asked about their sexual identity using a validated survey question from the Australian Study of Health and Relationships [35].

#### Fertility (young adult report)

Young adults were asked if they had ever been pregnant or ever tried to get pregnant (or get their partner pregnant if they were male). They were also asked about how long it took to achieve a pregnancy and whether this had resulted in the birth of a child/children.

#### Kessler psychological distress scale, ‘K10’ (young adult report)

The K10 is a scale for measuring psychological distress levels that may be associated with anxiety and affective disorders [36]. There are ten questions about negative emotional states experienced over the past four weeks. It has been validated as a screening instrument to identify likely cases of anxiety or depression in the community. Data items were coded and summed according to Kessler and raw scores were then transformed to a percentage score. Cases with missing values were excluded from the analysis [36].

#### Parental bonding instrument, ‘PBI’ (young adult report)

The PBI is a 25-item measure of parental bonding from the offspring perspective [37]. The level of care (emotional warmth versus coldness) and protectiveness (control versus autonomy granting) offspring perceive from each parent, and the combined effects of each dimension are assessed. Data items were coded, summed and scored according to prescribed methods to create a continuous score for ’care’ and for ‘protectiveness’ for each parent. Unknown values within each domain (don’t know, refused, missing) were imputed using horizontal mean imputation provided there were less than three missing items [29]. High/Low categorical variables were created from the continuous scale scores using the recommended cut-off scores for each parent [37]. A variable for each parent was then created from the combination of each parent’s two categorical results, assigning them to one of four groups including ‘high care and high protection’, ‘high care and low protection’, ‘high protection and low care’ and ‘low care and low protection’.

#### Relationship status – (young adult report)

Validated descriptive questions about family and social relationships were taken from the Australian Temperament Project [30].

#### Location of residence (maternal and young adult report)

The residential postal code for each participant was used to assign remoteness area codes to indicate if they reside in a metropolitan, regional or remote area [19].

#### Educational achievement (young adult report)

Young adults were asked to report their highest educational achievement. If they had completed year 12 (final year of secondary school), they were asked to report their Australian Tertiary Admission Rank (ATAR) [38]. The ATAR is a population based ranking of overall academic achievement. It is the primary criterion for entry into most undergraduate university programs throughout Australia, except Queensland. Results from students in Queensland were excluded due to use of a different academic measure and the degree of error in the process of conversion to the ATAR.

#### Occupation data (maternal and young adult report)

Occupations reported were classified using the Australian and New Zealand Standard Classification of Occupations used by the Australian Bureau of Statistics [39].

#### Disclosure of conception from ART (maternal and young adult report – ART group only)

Mothers were asked if they had disclosed their use of ART to conceive to their son or daughter, and if so, at what age. If they had not disclosed they were asked if they intended to disclose. Participating young adults were asked at what age they recalled being told of their ART conception.

### Addressing potential biases

#### Selection bias

Using ART medical record data it was possible to compare important characteristics, such as type of treatment and perinatal outcomes, between exposed maternal participants and non-participants. In the case of non-participating young adults, analysis of the maternal reports allowed differences in rates of birth defects, chronic illness and special health care needs to be examined in both the exposed and unexposed groups. Despite this, it is impossible to assess the degree of selection bias completely and outcomes will need to be interpreted with caution.

#### Recall bias

The study collected both concurrent and retrospective data with potential for recall bias. To minimise this, data were collected chronologically linking it to objective events including pregnancy and birth, first year of life, preschool, primary and secondary school periods. There were also questions that asked for comparison with others in the same age group, on the understanding that these would reveal major, clinically relevant concerns. It is still possible that the ART mothers may recall aspects of their offsprings’ health and wellbeing more vividly than the comparison group; this will be taken into account when interpreting results.

#### Reporting bias

There was potential for reporting bias with some of the more sensitive questions e.g. sexual development and sexual identity, however any such bias would likely be non-differential between exposed and unexposed groups. To minimize reporting bias, validated questions were used to assess sexual identity and sexual development - the Adolescence Scale includes both subjective and objective questions [34]. Potential reporting bias will be considered in the analysis of these outcomes.

#### Observer, inter-tester bias

This was minimised through use of a CATI and interviewer training to ensure a standardised, consistent approach. Data coding was conducted blinded to the mode of conception.

### Sample size

Sample size for the exposed group was based on the estimated ability to trace the mothers, the percent who would agree to participate and give permission for the researchers to contact their child and, finally, the percent of young adults who would agree to participate. To provide a best estimate, different scenarios were tested based on attrition rates at different stages for both mothers and young adults. For the primary outcome, young adult quality of life measured with the WHOQol-Bref, the following sample size calculations were made. Population norms were expected in the spontaneously conceived group with a mean of 72 and standard deviation of 18 for the Social domain. Therefore, using a two sample comparison of this mean, a sample size of 261 exposed and 261 unexposed young adults would detect an effect size of 0.25 as significant, and for a sample size of 424 in each comparison group, an effect size of 0.19 (Social Domain, alpha 0.05, power 80%) [40]. If an alpha of 0.01 was used for a same sample size of 261 for the Social domain, an effect size of 0.30 would be detected as significant or for a sample size of 424, an effect size of 0.24. Although there is no established cut point to indicate whether quality of life is unacceptably low or of ‘clinical significance’ with the WHOQol-Bref scale, an effect size of 0.25 would indicate that the score of the average person in the ART conceived group is lower than the scores of 60% of the control group. These were small to medium effect sizes and were associated with conservative estimates of sample size, based on the numbers available from the IVF centres.

### Statistical analysis

Descriptive analyses were conducted to obtain frequencies, means and standard deviations for variables of interest. These were followed by univariate analyses using statistics appropriate to the data distribution to assess whether exposures were significantly related to outcomes and to inform multivariate analyses. Multivariable models were built as appropriate with inclusion of other variables as potential confounders. Logistic regression models were built for binary outcome variables and linear regression models for continuous outcome variables. The presence of siblings in the exposed group was accounted for using general estimating equation regression methods. Potential confounders were selected for inclusion in regression models based on published literature or if the variable was associated with the outcome in univariable analysis at the p <0.1 level. 95% confidence intervals were calculated along with the p-values as significance estimates.

### Ethics

This study involved maternal and young adult populations from two health care services in the Victorian population. Following considerable consultation with key stakeholders to ensure the sensitivities of this field of research had been addressed, ethics approval was sought from and granted for all operations by: Royal Women’s Hospital – Project 08/37 and Epworth HealthCare – Project 46409. Ethics approval was also obtained from the Australian Institute of Health and Welfare – Project 2009/2/15, in relation to obtaining data from the National Death Index to determine maternal deaths.

## Discussion

World-wide, there are few existing studies of health outcomes in ART conceived offspring of this age group and sample size, and none of Australian populations [41,42]. We acknowledge that a retrospective cohort study design is less favourable than a prospective design due to potential recall bias, particularly when assessing areas such as growth and development. However, because we had a large cohort of ART conceived adults a retrospective approach maximised an existing opportunity.

In planning the study, many ethical considerations had to be addressed and most related to privacy. The potential implications for privacy of contacting women up to two decades after their attendance at an ART clinic and not knowing whether they had disclosed the use of ART to their child required sensitive strategies. Initial contact with the ART mothers was through the treating ART service rather than directly from the research team. Also, in case parents had not disclosed the method of conception to their son or daughter, young adults were only contacted with the consent of the mother.

Tracing a current maternal address (exposed group) from details provided so long ago, required multiple strategies, persistence and a substantial amount of time. Eventually 80% of mothers were successfully traced. The use of registered mail, where the addressee has to personally collect the letter at the post office, was essential to ensure receipt of the letter and maintain privacy. However, some stated that they had been alarmed at receiving registered mail as they had believed it may be a police or legal document.

Another challenge in the study design was finding an appropriate comparison group. The decision to use random digit dialing to recruit a spontaneously conceived group was pragmatic but also appropriate given that the aim of the study was to determine the health outcomes of ART conceived offspring rather than the causative effect of ART alone [17].

Many of the mothers who conceived with ART responded positively to the study invitation expressing their delight in having a child and their desire to assist the research. They were curious about potential health outcomes and interested in the findings. This was also the case with many mothers in the comparison group. A few mothers in the ART group (<1%) responded less favourably as they felt the study to be an invasion of their privacy or it reminded them of a difficult time in their life that they wanted to forget.

The responses to the study invitation from ART conceived young adults were also mostly positive but generally more ‘low key’ than that of their mothers. Although they were interested in the study findings, being ART conceived was often not particularly significant to them personally or something they had given a lot of thought to. Overall, this age group were very busy and finding time to take part was often a challenge. Despite this, when engaged at interview they were usually relaxed and willing to share information, both positive and negative.

The final reminder sent to non-responding mothers in the ART group was very important to the final response rate, increasing it by 11.6%. Many people later stated that they did not respond to the initial letter or the first reminder because they were busy or forgot, were going through a difficult time or lost the original pack. However, the last reminder provided impetus for a substantial number of women to respond. Consequently, we would recommend a ‘final reminder’ some months after the initial invitation to give respondents every opportunity to take part and increase response rate.

Dissemination of the study findings is critical, given the current paucity of knowledge in this area and the increase in demand for ART worldwide. The findings will be published in the peer-reviewed literature and utilised to inform educational materials about the long term health of ART conceived people. Study participants will also be provided with a summary of the key study findings. Finally, publication of the study protocol will assist others planning to follow-up ART populations as they reach adulthood.

## Competing interests

The authors declare that they have no competing interests.

## Authors’ contributions

All authors were involved in the project’s inception and or ongoing design and read, edited and approved the final manuscript.

## References

[B1] ESHRE ESHRE ART fact sheet Embryology ESoHRa, editor 2011 http://www.eshre.eu/ESHRE/English/Guidelines-Legal/ART-fact-sheet/page.aspx/1061. Accessed 1/08/2012, 2012

[B2] Halliday J Outcomes of IVF conceptions: are they different? Best Pract Res Clin Obstet Gynaecol 2007 21 1 67 81 10.1016/j.bpobgyn.2006.08.004 17055783

[B3] Basatemur E Sutcliffe A Follow-up of children born after ART Placenta 2008 29 Suppl B 135 140 18790325 10.1016/j.placenta.2008.08.013

[B4] Savage T Peek JC Robinson EM Ovarian stimulation leads to shorter stature in childhood Hum. Reprod Advance Access 2012 0 1 8 10.1093/humrep/des249 22777529

[B5] Carlijn G Vergouw E Kostelijk H The influence of the type of embryo culture medium on neonatal birthweight after single embryo transfer in IVF Hum Reprod Advance Access 2012 1 8 10.1093/humrep/des252 22791752

[B6] Maheshwari A Pandey S Shetty A Hamilton M Bhattacharya S Obstetric and perinatal outcomes in singleton pregnancies resulting from the transfer of frozen thawed versus fresh embryos generated through in vitro fertilization treatment: a systematic review and meta-analysis Fert Steril 2012 98 2 368 377 10.1016/j.fertnstert.2012.05.019 22698643

[B7] Van Balen F Development of IVF children Dev Rev 1998 18 30 46 10.1006/drev.1997.0446 11660550

[B8] McMahon C Gibson FL A special path to parenthood: parent-child relationships in families giving birth to singleton infants through IVF Reprod Biomed Online 2002 5 179 186 10.1016/S1472-6483(10)61622-7 12419044

[B9] Fisher JR Hammarberg K Baker HW Assisted conception is a risk factor for postnatal mood disturbance and early parenting difficulties Fertil Steril 2005 84 2 426 430 10.1016/j.fertnstert.2005.02.016 16084885

[B10] Wagenaar K Huisman J Cohen-Kettenis PT de Waal HA D-v An overview of studies on early development, cognition, and psychosocial well-being in children born after in vitro fertilization J Dev Behav Pediatr 2008 29 3 219 230 10.1097/DBP.0b013e318173a575 18550992

[B11] Golombok S MacCallum F Goodman E The “test-tube” generation: parent-child relationships and the psychological well-being of in vitro fertilization children at adolescence Child Dev 2001 72 2 599 608 10.1111/1467-8624.00299 11333087

[B12] Savage T Peek J Hofman PL Cutfield WS **Childhood outcomes of assisted reproductive technology** Hum Reprod 2011 26 9 2392 2400 10.1093/humrep/der212 21724570

[B13] Alukal JP Lipshultz LI Safety of assisted reproduction, assessed by risk of abnormalities in children born after use of in vitro fertilization techniques Nature clinical practice 2008 5 3 140 150 10.1038/ncpuro1045 18253110

[B14] Ceelen M Van Weissenbruch MM Vermeiden JP Van Leeuwen FE de Waal HA D-v Growth and development of children born after in vitro fertilization Fertil Steril 2008 90 5 1662 1673 10.1016/j.fertnstert.2007.09.005 18163998

[B15] Wilson CL Fisher JR Hammarberg K Amor DJ Halliday JL Looking downstream: a review of the literature on physical and psychosocial health outcomes in adolescents and young adults who were conceived by ART Hum Reprod 2011 26 5 1209 1219 10.1093/humrep/der041 21362683

[B16] Wang YA Chambers GM Sullivan EA Assisted reproductive technology in Australia and New Zealand 2008 2010 Canberra: Australian Institute of Health and Wellbeing

[B17] Carson C Kurinczuk JJ Sacker A Cognitive development following ART: effect of choice of comparison group, confounding and mediating factors Hum Reprod 2009 25 1 244 252 19828556 10.1093/humrep/dep344 PMC2794664

[B18] Fisher JR Hammarberg K Baker HG McBain JC Assessing the health and development of ART-conceived young adults: A study of feasibility, parent recall, and acceptability Reprod Heal 2008 5 7 10.1186/1742-4755-5-7 PMC2583986 18957131

[B19] Australian Bureau of Statistics ASGC remoteness area classification: purpose and Use 2001 Canberra: Commonwealth of Australia

[B20] Roberts CL Lancaster PA Australian national birthweight percentiles by gestational age Med J Aust 1999 170 3 114 118 10065122 10.5694/j.1326-5377.1999.tb127678.x

[B21] Riley M Halliday J Birth defects in Victoria 2005-2006 2008 Melbourne: Victorian Government Department of Human Services

[B22] World Health Organisation International statistical classification of diseases and related health problems 10th revision. 2010; Version 10 onlinehttp://apps.who.int/classifications/icd10/browse/2010/en. Accessed 1/08/2012

[B23] Waters E Salmon L Wake M Hesketh K Wright M The parent-form child health questionnaire in Australia: comparison of reliability, validity, structure and norms J Pediatr Psychol 2000 25 381 391 10.1093/jpepsy/25.6.381 10980043

[B24] Waters E Salmon L Wake M Hesketh K Wright M The child health questionnaire in Australia: reliability, validity and population means Aust N Z J Public Health 2000 24 2 207 210 10.1111/j.1467-842X.2000.tb00145.x 10790944

[B25] Bethell CD Read D Stein RE Blumberg SJ Wells N Newacheck PW Identifying children with special health care needs: development and evaluation of a short screening instrument Ambul Pediatr 2002 2 1 38 48 10.1367/1539-4409(2002)002<0038:ICWSHC>2.0.CO;2 11888437

[B26] CAHMI National survey of children with special health care needs. NS-CSHCN 2005/2006 2011 http://childhealthdata.org/learn/NS-CSHCN. Accessed 7/06/2011, 2011

[B27] The WHOQoL Group Development of the world health organisation WHOQoL-BREF quality of life assessment Psychol Med1998b 28 551 558 9626712 10.1017/s0033291798006667

[B28] Murphy B Herrman H Hawthorne G Pinzone T Evert H Autralian WHOQOL instruments: User’s manual and interpretation guide Centre WFS, ed 2000 Melbourne: University of Melbourne http://www.psychiatry.unimelb.edu.au/centres-units/cpro/whoqol/instruments/manual.pdf. Accessed 22/06/2009

[B29] Hawthorne G Elliott P Imputing cross-sectional missing data: comparison of common techniques Aust N Z J Psychiatry 2005 39 583 590 10.1080/j.1440-1614.2005.01630.x 15996139

[B30] Australian Institute of Health and Welfare Making progress: the health, development and wellbeing of Australia’s children and young people 2008 Canberra: Australian Government

[B31] Cole T Feeman J Preece M Body-mass index reference curves for the UK, 1990 Arch Dis Child 1995 73 25 29 10.1136/adc.73.1.25 7639544 PMC1511150

[B32] World Health Organisation Obesity: preventing and managing the global epidemic 2000 Geneva: Department of Nutrition and Health Development 11234459

[B33] Gruzelier JH Kaiser J Syndromes of schitzotypy and timing of puberty Schizoph Res 1996 21 183 194 10.1016/0920-9964(96)00050-3 8885046

[B34] Kaiser J Gruzelier JH The Adolescence Scale (AS-ICSM): a tool for the retrospective assessment of puberty milestones Acta Paediatr Suppl 1999 88 429 64 68 10419234 10.1080/080352599750029943

[B35] Smith AM Rissel CE Richters J Grulich AE de Visser RO Sex in Australia: sexual identity, sexual attraction and sexual experience among a representative sample of adults Aust N Z J Public Health 2003 27 2 138 145 10.1111/j.1467-842X.2003.tb00801.x 14696704

[B36] Kessler RC Andrews G Colpe LJ Short screening scales to monitor population prevalences and trends in non-specific psychological distress Psychol Med 2002 32 6 959 976 10.1017/S0033291702006074 12214795

[B37] Parker G Tupling H Brown LB A parental bonding instrument Br J Medical Psychology 1979 52 1 10 10.1111/j.2044-8341.1979.tb02487.x

[B38] Wikipaedia Australian Tertiary Admissions Rank http://en.wikipedia.org/wiki/Australian_Tertiary_Admission_Rank. Accessed 1/08/2012

[B39] Trewin D Pink B Australian and New zealand standard classification of occupations Statistics ABo, ed 2006 First edition Canberra: Australian Bureau of Statistics

[B40] Stata statistical software: release 10.1 [computer program] 2007 College Station, TX: StataCorp

[B41] Beydoun H Sicignano N Beydoun M A cross-sectional evaluation of the first cohort of young adults conceived by in vitro fertilization in the United States Fertil Steril 2010 94 6 2043 2049 10.1016/j.fertnstert.2009.12.023 20159654 PMC2955173

[B42] Owen L Golombok S Families created by assisted reproduction: parent-child relationships in late adolescence J Adolesc 2009 32 835 848 10.1016/j.adolescence.2008.10.008 19010525

